# Lifestyle Interventions with a Focus on Nutritional Strategies to Increase Cardiorespiratory Fitness in Chronic Obstructive Pulmonary Disease, Heart Failure, Obesity, Sarcopenia, and Frailty

**DOI:** 10.3390/nu11122849

**Published:** 2019-11-21

**Authors:** Hayley E. Billingsley, Paula Rodriguez-Miguelez, Marco Giuseppe Del Buono, Antonio Abbate, Carl J. Lavie, Salvatore Carbone

**Affiliations:** 1 Department of Internal Medicine, VCU Pauley Heart Center, Virginia Commonwealth University, Richmond, VA 23284, USA; hayley.billingsley@vcuhealth.org (H.E.B.); antonio.abbate@vcuhealth.org (A.A.); 2 Department of Kinesiology & Health Sciences, College of Humanities & Sciences, Virginia Commonwealth University, Richmond, VA 23284, USA; prodriguezmig@vcu.edu; 3 Department of Cardiovascular and Thoracic Sciences, Catholic University of the Sacred Heart, 00168 Rome, Italy; marcodelbuono@hotmail.it; 4 Department of Cardiovascular Diseases, Ochsner Clinical School, New Orleans, LA 70121, USA; clavie@ochsner.org

**Keywords:** cardiorespiratory fitness, peak oxygen consumption, cardiopulmonary exercise testing, chronic obstructive pulmonary disease, heart failure, obesity, sarcopenia, frailty

## Abstract

Cardiorespiratory fitness (CRF) is an independent predictor for all-cause and disease-specific morbidity and mortality. CRF is a modifiable risk factor, and exercise training and increased physical activity, as well as targeted medical therapies, can improve CRF. Although nutrition is a modifiable risk factor for chronic noncommunicable diseases, little is known about the effect of dietary patterns and specific nutrients on modifying CRF. This review focuses specifically on trials that implemented dietary supplementation, modified dietary pattern, or enacted caloric restriction, with and without exercise training interventions, and subsequently measured the effect on peak oxygen consumption (VO_2_) or surrogate measures of CRF and functional capacity. Populations selected for this review are those recognized to have a reduced CRF, such as chronic obstructive pulmonary disease, heart failure, obesity, sarcopenia, and frailty. We then summarize the state of existing knowledge and explore future directions of study in disease states recently recognized to have an abnormal CRF.

## 1. Introduction

Cardiorespiratory fitness (CRF), often expressed as maximal oxygen consumption (VO_2_ max) in healthy individuals or peak VO_2_ in those with limitations to exercise, reflects the maximal amount of oxygen that can be taken up, perfused and transported in the blood, absorbed, and utilized during strenuous exercise [1]. The gold standard of VO_2_ measurement is cardiopulmonary exercise testing (CPET), as it allows for the evaluation of the cardiovascular (CV), respiratory, and muscular responses to maximal exercise encompassed under the term CRF [2]. Recently, CRF has been proposed as a vital sign due to its strong inverse association with all-cause and CV disease (CVD) mortality, which is stronger than many traditional risk factors such as type 2 diabetes mellitus (T2DM) and smoking [1,3], and independent of sex and race [4]. While CRF is best established as a predictor of all-cause- and CVD-specific mortality, it has also been associated with decreased mortality from cancer [5,6]. Importantly, improvements or maintenance in CRF over time are associated with lowered all-cause mortality risk [7]; even in older adults, those with the highest CRF have the lowest risk of all-cause mortality [3]. In adulthood, however, there is an average physiologic decline in VO_2_ max of about 10% per decade, regardless of physical activity (PA) level [8]. Notably, PA also decreases with age, especially vigorous activity capable of modulating CRF [9]. Therefore, achieving and maintaining the highest level of CRF possible in early adulthood is important for lowering mortality risk in all individuals, even in those classified as low risk for CVD [4,10].

Importantly, although CRF is determined by many factors that cannot be modified, such as age, sex, and underlying disease state(s), in many instances, CRF remains a modifiable risk factor [11]. Exercise training (ET), defined as repeated, structured activity to maintain or increase physical fitness, and increased PA, which encompasses all bodily movement performed by skeletal muscles (SM) resulting in movement, are indeed the most established methods of increasing CRF in apparently healthy individuals [12], as well as those with noncommunicable diseases [13,14,15,16]. The use of some medications (e.g., renin–angiotensin–aldosterone system inhibitors, hydralazine, digoxin, ivabradine) in populations with impaired CRF, such as heart failure (HF), has also led to increases in peak VO_2_ [2,17]. Less is known about nutritional strategies to improve CRF; however, dietary interventions have the ability to modify and improve other CV and metabolic risk factors for mortality, such as body weight [18], body composition [19], blood pressure (BP) [20], lipids [21], and inflammation [22], as well as hard endpoints, such as incidence of T2DM [23], CVD [24], and breast cancer [25]. In this review, we explore the effects of nutritional strategies to improve CRF in patients with chronic diseases, with a focus on chronic obstructive pulmonary disease (COPD), HF, obesity, sarcopenia, and frailty. Sarcopenia, obesity, and their combined presence referred to as sarcopenic obesity are body composition abnormalities associated with an impairment in CRF [26]. While obesity refers to an increased amount of fat mass (FM) that affects health, sarcopenia is a progressive loss of SM mass (SMM), as well as strength and functionality of this tissue [27]. These noncommunicable disease states were selected, as CPET and surrogate field tests estimating CRF have not routinely been included as part of clinical and research assessment in all chronic disease populations, especially in regards to nutrition interventions. Finally, emerging populations that may benefit from nutrition intervention on CRF and future directions are discussed.

## 2. Chronic Obstructive Pulmonary Disease

COPD is a life-threatening lung disorder associated with airflow obstruction, exercise intolerance, and overall impairment of health. Disabling symptoms, including dyspnea and fatigue, and lower exercise capacity have been associated with a higher risk of mortality in individuals with COPD [28]. The mechanisms behind exercise intolerance in this population are, however, not fully understood due to the multifactorial mechanisms that contribute to it. Pulmonary limitations caused by poor elastic recoil, increased airways resistance, increased pulmonary vascular resistance, endothelial dysfunction, impaired cardiac output, peripheral muscle dysfunction, and poor nutritional status seem to contribute to this pathological process in a different fashion. Thus, patients often complain of exertional dyspnea, fatigue, muscle weakness, and leg fatigue. Pulmonary rehabilitation (PR) represents a comprehensive intervention that combines ET and disease management and is commonly used to improve patients’ physical and psychological condition [29]. Benefits of PR specifically associated with exercise intolerance include increased CRF, improved muscle strength and endurance, as well as reduced symptoms of dyspnea [29]. The implementation of PR in the post-hospitalization period not only reduces the risk of rehospitalization but also mortality [30].

### 2.1. Assessment of CRF in COPD

Considering that the assessment of exercise capacity through CPET is not widely available and may be intimidating to some patients, other field tests have been developed for an easier and more accessible measure of functionality in certain populations. One specific example is the 6-min walk test (6MWT), which is predictive of mortality in COPD patients. This test is considered a good assessment of functional capacity, although there is an acknowledged learning effect that needs to be considered when interpreting the results [31,32]. Notably, the 6MWT, as other walking tests, does not provide insights on the determinants responsible for the functional limitation (i.e., CV vs. pulmonary vs. SM), which can, in fact, be determined with CPET. Similarly, the incremental shuttle walking test (ISWT) measures distance walked and correlates well with peak VO_2_ in COPD, predicting survival and hospital re-admission in this population [33]. Lastly, the endurance shuttle walking test (ESWT) also evaluates endurance capacity and has been proposed as another tool to evaluate CRF in COPD [33]. However, some limitations have been described with this test, and its use has not been validated yet. Nutrition interventions aimed at improving exercise tolerance are often combined with PR with the intention of increasing the effects of ET on CRF [34]. A variety of nutritional adjuncts to PR have been studied, including creatine [35,36,37], omega-3 (*N*-3) polyunsaturated fatty acids (PUFA) [38], dietary nitrates [39,40,41], micronutrient supplementation [42,43,44], and nutrition support supplementation [45,46,47,48,49,50]. Notably, the specifics of ET in combined nutrition and PR interventions are often vaguely specified outside of modality of ET, frequency, and total time of visits.

### 2.2. Creatine Supplementation in COPD

Dietary supplementation with creatine monohydrate has been used to enhance the response to high-intensity ET in healthy populations [51]. Creatine monohydrate increases the amount of phosphorylated creatine (PCr) available for ATP synthesis during strenuous exercise and contributes to PCr resynthesis [35]. Importantly, PCr content has been demonstrated to be lower in subjects with COPD than healthy age-matched subjects [52]. In a randomized, double-blind placebo-controlled trial combining PR and creatine supplementation in patients with COPD (mild-to-moderate severity), creatine has not shown any modulation on CRF defined as changes in peak VO_2_ or distance walked on ISWT [35,36]. Creatine supplementation combined with PR has also failed to improve aerobic endurance over placebo measured by the ESWT [37]. There have been mixed reports regarding the effect of creatine supplementation combined with PR on muscle strength and endurance in patients with COPD [35,36]. Fat-free mass (FFM), a surrogate measure of SMM and a major determinant of CRF [27,53], was increased with creatinine supplementation and PR [35,36]. This may, however, be due to intramuscular fluid retention, which would be read as an increase in FFM, and not necessarily SMM [35,36]. Similar results have been previously described in healthy elderly individuals undergoing dietary creatine supplementation and resistance ET, with associated improvements in FFM and isometric strength, but not functional capacity [54]. In conclusion, creatinine does not seem to significantly modulate CRF in individuals with COPD. Further exploration of varying doses and length of treatment of creatinine on muscle strength and endurance, as well as on FFM, is, however, warranted.

### 2.3. Polyunsaturated Fatty Acid Supplementation in COPD

Dietary intake of *N*-3 PUFA has been proposed to promote anti-inflammatory benefits in patients with COPD [55]. Despite these promising results, intake of *N*-3 PUFA has failed to increase CRF in individuals with COPD (moderate severity), but has augmented muscle function. An 8-week randomized placebo-controlled trial examining *N*-3 PUFA supplementation in patients undergoing inpatient PR failed to show an improvement on peak VO_2_ [38]. The N-3 PUFA supplement consisted of 340 mg docosahexanoic acid (DHA) (22:6*n*-3), 700 mg eicosapentanoic acid (EPA) (20:5*n*-3), 1200 mg alpha-linolenic acid (ALA) (18:3*n*-3), 760 mg gamma-linolenic acid (18:3*n*-6), and 400 mg stearidonic acid (18:4*n*-3) [38]. The placebo supplement was isocaloric and contained a blend of 80% palm and 20% sunflower oils [38]. Submaximal exercise time, peak workload, and mechanical efficiency (ratio of VO_2_ to peak work load) were, however, increased in the *N*-3 PUFA group compared with placebo [38]. The authors also observed an increase in respiratory exchange ratio (RER) in the group under the *N*-3 PUFA supplementation when compared with those receiving placebo, which they hypothesized may be due to an ability to go further into anaerobic metabolism, but without providing more information about potential mechanisms [38]. Thus, the impact of *N*-3 PUFA supplementation to improve CRF in COPD merits further study.

### 2.4. Micronutrient Supplementation in COPD

Malnutrition is commonly observed in patients with COPD and is associated with hypovitaminosis, especially vitamin D deficiency [56]. Although the precise role that vitamin D may exert on the pathophysiology of COPD is not completely understood, several studies have suggested involvement in airway impairment and exacerbated inflammatory response [57]. In addition, vitamin D deficiency may also be associated with impaired SM function, playing a potential role in CRF [42]. Indeed, patients with lower concentrations of vitamin D have reported shorter distance in the 6MWT than those with higher circulating content of this antioxidant [57]. Although these relationships infer promising results using vitamin D supplementation in COPD, a limited number of clinical trials have explored this possibility, and results obtained are conflicting. A randomized, double-blind, placebo-controlled trial in a very severe cohort of patients with COPD showed that supplementation with 100,000 IU of vitamin D for one year was ineffective to reduce pulmonary exacerbations [43]. A post-hoc subgroup analysis of the data, however, suggested that participants participating in PR and receiving vitamin D supplementation exhibited greater improvements in muscle strength of inspiratory muscles and overall oxygen uptake when compared to those not participating in PR [42].

Similarly, supplementation with vitamin B12, a complex involved in mitochondrial metabolism and exercise tolerance [58,59], has been explored in COPD with positive results. A randomized, double-blind clinical trial used PR in combination with 8 weeks of 500 mg of daily B12 supplementation in subjects with moderate-to-severe COPD [44]. Although authors described discrete positive effects on exercise tolerance evaluated through CPET, no significant changes were observed in oxygen consumption between groups, with the exception of improved total exercise time [44]. The authors noted that small sample size and disparity in baseline physical activity between participants may have limited trial results [44]. Further studies are needed to elucidate the potential impact of vitamin supplementation alone or in combination with PR on CRF of individuals with COPD.

### 2.5. Dietary Nitrate Supplementation in COPD

Nitric oxide (NO) is a critical vasodilator involved in blood flow regulation and oxygen (O_2_) delivery to SM [39]. Although airflow obstruction may be involved in exercise intolerance in COPD, other mechanisms, including vascular dysfunction and associated low NO bioavailability, play critical roles in this manifestation [39]. Dietary nitrates have the potential to increase NO, increasing the supply and delivery of O_2_ to the working SM, and thus improving CRF [39]. Multiple studies have described that beetroot juice, a major dietary source of nitrates, enhances NO synthesis, vascular function, and exercise capacity in healthy individuals [60,61,62]. Despite the compelling observations in multiple populations, results in COPD are unclear. Positive results have been described in eleven subjects with COPD (moderate-to-very severe) that consumed an acute dose of beetroot juice or placebo [39]. Three hours post-dose, nine participants exhibited an improved ISWT when compared with placebo [39]. Although the study was completed in a small and heterogeneous population, authors concluded that dietary nitrate supplementation abrogated exercise-related fatigue in patients with COPD [39]. Another study also described that an acute ingestion of beetroot juice mildly extends the time to fatigue during submaximal exercise in COPD (mild-to-moderate severity) [63]. Conversely, in a different study, after a 6-day trial of beetroot juice consumption, distance on the 6MWT did not improve [40]. Similar results were observed in another acute dietary nitrate supplementation intervention that described similar ESWT distance and time to fatigue between active and placebo in the same population [64]. Notably, longer supplementation with beetroot juice (8 days) showed similar results with no improvements in peak VO_2_ [41]. The authors surmised that dietary nitrate supplementation in COPD may be inconsistent due to the inter-subject variability in age, disease severity, and comorbidities. None of the above studies included PR or ET as part of the intervention. In addition, the authors also highlighted the multiple mechanisms of dysfunction contributing to reduced CRF in COPD that may also limit the response observed [41]. Other potential factors contributing to the disparity in results may be associated with medication. Beta 2 adrenergic receptor agonists, one of the most common treatments for COPD, are known to increase endothelial NO synthesis and may attenuate the potential effects of dietary nitrate supplementation [65]. In addition, other medications, including ACEi/ARBs, have been proposed as potential pharmacologic interventions that alter NO bioavailability [66], though other results conflict with this finding [67].

### 2.6. Nutrition Support Supplementation in COPD

Malnutrition further aggravates exercise intolerance in COPD. Specially, these individuals are at high risk for loss of SMM and the development of sarcopenia and cachexia [68]. Thus, the combination of calorically-rich supplement strategies with PR has been proposed to improve CRF in this population. Overall, nutritional support promotes an increase in weight gain, muscle strength, pulmonary function, exercise capacity, and overall quality of life in malnourished patients with COPD [69,70]. Respifor is a dietary supplement that affects CRF and body composition [45,50]. Respifor contains 60% carbohydrate, 20% fat, 20% protein, and provides 570 calories when taken orally three times a day [45,50]. Its effects on CRF and body composition have been studied as part of the INTERCOM trial in muscle-wasted patients (defined by unintentional weight loss or reduced FFM index (FFMI = FFM kg/ht^2^)) [50]. Thirty-nine patients were randomized to usual care or Respifor supplementation for a period of 4 months in combination with ET, proceeded by 20 months of active maintenance [50]. Active maintenance included nutrition supplementation, if still indicated, as well as less frequent visits with a dietitian and exercise physiologist to measure adherence [50]. After 4 months, the intervention group demonstrated a significant gain in FFMI, as well as an increase in functional capacity measured by 6MWT distance and peak watts achieved on cycle ergometer over the usual care group [50]. At 24 months, cycle ergometry was not repeated, but 6MWT distance was significantly reduced in the usual care group versus the intervention group, which remained stable from baseline, suggesting a protective effect of the supplement by preventing the decline in functional capacity [50]. Interestingly, an earlier randomized placebo-controlled trial examined an equal amount of Respifor supplementation in a group of patients referred for a 7-week PR program. This study found that while ISWT did not change in the overall cohort (*N* = 60 completed), when patients with a body mass index (BMI) < 19.0 kg/m^2^ were excluded (*N* = 8), ISWT significantly increased in supplemented patients versus controls [45]. These conflicting results could possibly be due to the length of the PR, in combination with the supplementation intervention, which was significantly longer in the INTERCOM study [45,50]. In addition, weight gain was also significant only in the supplemented patients with BMI >19 kg/m^2^, but this was driven by an increase in FM [45]. Interestingly, previous studies with other nutritional supplements (i.e., beetroot juice) have also denoted a disparity between responders and non-responders based on BMI, inferring a potential role of adiposity that needs to be further explored [71]. Additionally, a study of 36 individuals with COPD (moderate-to-severe), who were less than ≤110% of ideal body weight, demonstrated benefit in a 12-week home-based PR and nutrition supplementation (200 calories, 55% carbohydrate, 20% fat, 25% protein incorporating whey peptides, *N*-3 PUFA consisting of 4 g ALA [18:3*n*-3], 800 mg DHA [22:6*n*-3], 1.2 g EPA [20:5*n*-3], and vitamins A, C, and E taken twice daily). The authors described improvements in 6MWT distance and weight, specifically FM, that increased significantly in the group receiving PR in combination with the supplement compared with the control [47]. Biomarkers of systemic inflammation, such as high-sensitivity C-Reactive Protein (CRP) and Tumor Necrosis Factor Alpha (TNFα), also decreased significantly in patients receiving PR and nutritional supplement compared with the control. The authors attributed these results to the anti-inflammatory effects of whey peptide; however, such a hypothesis would clearly require further confirmatory studies [47]. Furthermore, as there was no exercise-only group in this study, it is difficult to separate the effects of supplementation versus that of PR [47]. Another study utilizing an energy-containing supplement in individuals with COPD and evidence of malnutrition (i.e., low FFMI, BMI, or evidence of weight loss) demonstrated that oral nutrition supplements (83% carbohydrate, 30% fat, 17% protein [no calorie amount given]) in combination with PR was not more effective than PR alone in increasing 6MWT or ISWT distance [49]. Patients with nutrition supplementation, in addition to PR, did increase weight and FFMI to a greater degree than PR alone [49]. Lastly, the NUTRAIN trial, a recent double-blind randomized controlled trial, looked at the effects of a supplement providing 188 calories, 60% carbohydrate, 20% fat, and 20% protein, and enriched with *N*-3 PUFA (consisting of 367.9 mg ALA [18:3*n*-3], 118.5 mg DHA [22:6*n*-3], and 248.8 mg EPA [20:5*n*-3]), leucine, and vitamin D in individuals with COPD and low FFMI in addition to 4 months of PR [46]. Post-intervention, body weight and FM were significantly higher in patients taking supplement versus control, but 6MWT distance was not different [46]. Greater carbohydrate content mainly served to increase supplemented calories. Nonetheless, a greater caloric intake through higher content in fats has been proposed as a more beneficial approach for some patients with COPD, especially those with hypercapnia and severe shortness of breath [72,73,74,75]. The reason behind this recommendation relies on the metabolism of fats that produces less carbon dioxide (CO_2_) per O_2_, resulting in a lower RER compared with carbohydrate metabolism, and therefore, will not increase CO_2_ content in hypercapnic patients. Despite these possible benefits, further studies are needed to determine if nutritional supplements rich in fats will improve CRF in patients with COPD.

### 2.7. Summary of Nutrition Interventions in COPD

In summary, while PR demonstrates a clear benefit for individuals with COPD, no effective adjunct nutrition strategy for enhancement of CRF has been identified at this time. While creatine and dietary nitrates are not likely a useful strategy for CRF enhancement [35,36,37,39,40,41], some trials with high energy supplements have shown a potential benefit [45,47,50], while others have not [46,49] (Table 1). Additionally, *N*-3 PUFA may have also demonstrated some benefit on enhancing exercise capacity, though a direct benefit on peak VO_2_ was not shown [38]. We also believe that future work should more clearly describe the ET component utilized in PR, the timing of the nutrient supplement in relation to exercise, and, when possible, utilizing objective biomarkers of nutrient consumption. Furthermore, considering the heterogeneity of COPD pathophysiology, we recommend that a greater description of the disease severity, including disease severity stage, medication, and comorbidities, as well as weight and muscle wasting status, may help elucidating which sub-group of patients with COPD may benefit the most from dietary supplementation.

## 3. Heart Failure

The clinical syndrome of HF is characterized by reduced CRF accompanied by dyspnea and fatigue [2]. Low CRF is a significant risk for HF, and improvements in peak VO_2_ through ET are associated with decreases in CVD and all-cause mortality, as well as HF hospitalizations [100,101]. A reduction in cardiac output is a major contributor to reduced CRF in HF patients, but individuals with HF also suffer malfunction throughout the oxygen transport chain in the form of pulmonary and SM abnormalities, as well as other exercise-limiting comorbidities such as obesity and anemia [2]. There are two major types of HF—HF with reduced ejection fraction (HFrEF) and HF with preserved ejection fraction (HFpEF). Although individuals with HFrEF and HFpEF both suffer from exercise intolerance, individuals with HFrEF may be more limited by central reductions in cardiac reserve, while those with HFpEF may be more limited by SM abnormalities in oxygen extraction and utilization [102], as well as other comorbidities such as obesity [103,104]. The majority of studies examining nutrition strategies to increase CRF have been performed in HFrEF patients, and there exists a major need to address the lack of therapies available to HFpEF patients.

Nutrition interventions to improve CRF in HFrEF patients have included micronutrient supplementation [76], amino acids (AA) [77,78,79,80,81], nutrition support supplementation [84], *N*-3 PUFA [82,83], and dietary nitrates [85,86].

### 3.1. Micronutrient Supplementation in HFrEF

Micronutrient supplementation was explored as a potential therapy to increase CRF, as patients with heart failure may be more susceptible to multiple deficiencies due low dietary intake, altered absorption, and pharmacologic treatments, and may have increased needs due to increased oxidative stress, as well as dysfunction of SMM and myocardial tissue [76]. In a double-blind, randomized controlled trial with 32 older adults (>70 years) participants with HFrEF due to ischemic cardiomyopathy receiving a daily micronutrient supplement containing calcium, magnesium, zinc, copper, selenium, B-vitamins, vitamins A,C,D, and E, as well as co-enzyme Q10 or placebo for 9 months [76], patients receiving the supplement experienced positive cardiac remodeling compared to placebo, with reduced left ventricular (LV) end-diastolic and end-systolic volumes and improved LVEF measured by cardiac magnetic resonance imaging (MRI). There was no ET included as part of the intervention [76]. No changes in inflammatory biomarkers or 6MWT distance in supplemented patients versus controls were found [76]. Additionally, small trials in HFrEF patients have shown that thiamine supplementation improves LVEF and may also provide symptomatic improvements in these individuals; however, given the lack of good quality data, results should be interpreted cautiously [105].

### 3.2. Amino Acid Supplementation in HFrEF

Supplementation of multiple or individual AA have demonstrated mixed results on modulation of CRF in individuals with HFrEF. AA can be utilized as energy-producing substrates in the citric acid cycle, and SM of individuals with HF have demonstrated increased AA metabolism at rest. A group of 95 older (>65 years of age) HFrEF patients with reduced exercise tolerance, defined as a peak VO_2_ < 15 mL·kg^−1^·min^−1^, was supplemented with 4 g of essential AA twice a day or an isocaloric placebo composed of carbohydrate in a 30-day randomized double-blinded control trial [78]. After 30 days, participants in the AA group demonstrated increased relative peak VO_2_ on cycle ergometer CPET versus placebo [78]. Similarly, it was demonstrated in another randomized control trial that 2 months of supplementation with 8 g of essential AA increased peak VO_2_ and 6MWT distance, as well as body weight in patients with adequate protein-energy intake at baseline and without depletion of SMM defined by reduced arm muscle area [79]. In a pilot-trial of 13 HFrEF patients with dilated cardiomyopathy, a mix of essential and semi-essential AA was administered at the dose of 4 g twice daily for 3 months [77]. After 3 months, relative peak VO_2_ on cycle ergometer CPET was increased from baseline, as was VO_2_ at the anaerobic threshold. Distance on the 6MWT was also improved [77]. There was no change in echocardiographic parameters, but a trend in reduction of NT-proBNP was observed [77]. Not all AA supplements have proved successful however. In a randomized control trial of 66 individuals with HFrEF, subjects were assigned to either 5 g of branched chain AA (BCAA) twice a day plus resistance ET or resistance ET alone for 3 months [80]. Resistance ET sessions were performed for 1 h twice a week and in the supplementation group, 5 g of the BCAA was administered immediately prior to exercise [80]. An individualized, standard diet was prescribed to all participants, and the supplemented group was instructed to subtract 10 g of protein from their diet to account for the additional intake through the BCAA supplement [80]. At 3 months, there were increases in peak VO_2_ (estimated from metabolic equivalent of tasks [METs] achieved on the treadmill stress test) and muscular strength measured by handgrip dynamometer in both groups, with no differences between the groups [80]. Phase angle, a measure of impedance gathered by bioelectrical impedance analysis which helps characterize quality and quantity of soft tissue [106], also increased in both groups, without a difference between groups [80]. Dietary intake, including protein intake, measured by 24-h dietary recall was also not different between groups at 3 months [80]. The authors proposed that the pre-existing pro-inflammatory, catabolic state in patients with HF may have induced a state of malabsorption in which patients could not utilize the BCAA for energy-producing purposes in the citric acid cycle or anabolism [80]. In contrast, the aforementioned trials using essential AA have suggested that these supplements can in fact enhance CRF, making it likely that the supplements were absorbed and utilized by the body [77,78,79]. Only one of these trials utilized a biomarker to objectively measure intake (leucine), showing a significant increase in plasma leucine after supplementation [79]. Additionally, only one of the trials included ET as part of the intervention [80].

**Figure 1:**
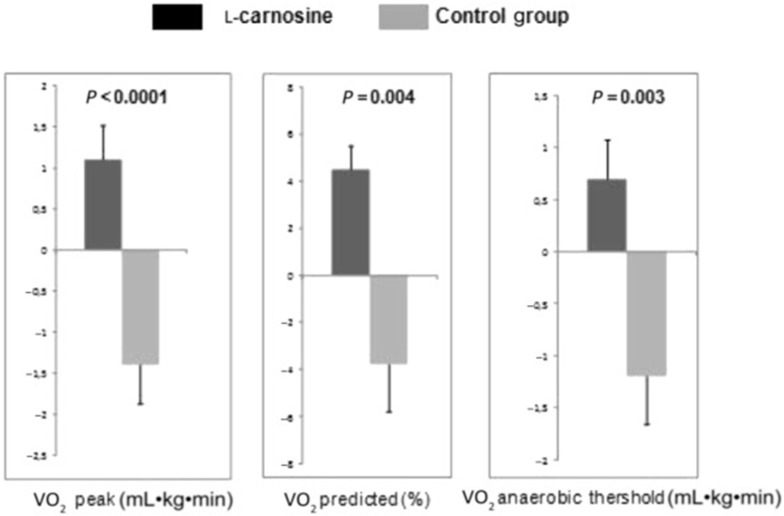
Six months of supplementation with 500 mg L-Carnosine daily increased VO_2_ (mL/kg/min), percent predicted peak VO_2_(%), and VO_2_ at the anaerobic threshold (mL/kg/min) versus standard treatment in 50 patients with HFrEF. Used with permission by Lombardi et al. [81].

Levo-carnosine (L-carnosine), a dipeptide composed of beta-alanine and histidine, has also been examined for its effects on CRF. L-Carnosine is a multi-functional molecule that functions as an antioxidant and declines with aging, shown to be potentially cardio- and nephro-protective, and helps buffer lactic acid during physical exertion [81]. A randomized control trial of 50 individuals with HFrEF examined the effects of 500 mg daily of L-carnosine versus standard care for 6 months [81]. There was no ET included as part of the intervention [81]. At 6 months, no within-group statistical changes were reported with regards to exercise variables, with the exception of the 6MWT, which was improved in the L-carnosine group, but not in the control group [81]. However, the changes in peak VO_2_ were significantly different between the L-carnosine group (+1.1 mL/kg/min) compared to the control, which, in fact, experienced a trend toward a reduction in peak VO_2_ (−1.4 mL/kg/min). Similarly, the changes of VO_2_ at the anaerobic threshold, a measure of submaximal effort [2] and peak ET workload on cycle ergometer CPET, favored the L-carnosine-supplemented group versus control (Figure 1) [81]. Of note, no significant changes in echocardiographic parameters occurred [81]. Because there were no significant changes in the primary analysis of the study in the within-group analysis, the potential benefits described should be interpreted with caution, and clearly require further study in an appropriately powered randomized controlled trial.

The authors surmised that the observed positive effects of L-carnosine on CRF were very likely due to the pH buffering effect, which delayed muscular acidosis [81].

### 3.3. Polyunsaturated Fatty Acid Supplementation in HFrEF

Glutamine, an AA involved in the production of the endogenous antioxidant glutathione, as well as decreasing muscular proteolysis, has been combined with *N*-3 PUFA to examine the effects on CRF in HFrEF. In a double-blind, randomized placebo-controlled trial, 31 subjects with HFrEF were assigned to receive either a combination supplement of 6.5 g of *N*-3 PUFA and 8 g of glutamine or an isocaloric placebo composed of safflower oil and milk powder for 3 months [82]. No ET was included as part of the intervention. Although LM increased in the active group upon dual-energy X-ray absorptiometry (DEXA) scan, there were no differences on exercise parameters on cycle ergometer CPET, 6MWT distance, measures of muscular strength, echocardiographic parameters, or SM biopsy [82]. In contrast, another trial of *N*-3 PUFA supplementation without glutamine demonstrated an increase in CRF with *N*-3 PUFA supplementation [83]. A randomized, double-blind placebo-controlled trial assigned 133 individuals with HFrEF of non-ischemic etiology to 12 months of placebo or *N*-3 PUFA supplementation, beginning with a 1 g gelatin capsule containing 850–882 mg *N*-3 PUFA capsules 5 times a day for the first month, decreasing to 2 daily capsules for the remaining 11 months [83]. There was no ET included as part of the intervention [83]. At the 12-month follow-up, LVEF was significantly increased, while LV diameters and volumes were significantly decreased in the *N*-3 PUFA-supplemented patients versus the placebo group [83]. In *N*-3 PUFA-supplemented patients, relative peak VO_2_, workload, and length of ET were increased relative to the placebo group [83]. Additionally, inflammatory biomarkers, including TNFαa, interleukin-6 (IL-6), and IL-1, were decreased significantly in supplemented patients versus placebo [83]. The authors attributed the increase in CRF to the observed increases in cardiac functionality, which they theorized was improved by multiple factors, including the reduction in inflammatory biomarkers, as well as improved contractile and endothelial functioning [83]. Interestingly, however, a decrease in diastolic function defined as an increased deceleration time was also observed in the supplemented subjects versus placebo [83].

### 3.4. Nutrition Support Supplementation in HFrEF

Cardiac cachexia, or the concomitant loss of lean mass (LM) and FM, in advanced HF patients is linked to poor prognosis [27,107]. Gaps in understanding continue to exist, but multiple mechanisms are likely at play, including high levels of inflammation, alterations in hunger/satiety signaling, changes in energy metabolism, as well as poor perfusion leading to malabsorption in the gut [107]. Though low intake alone is not causative, nutrition supplements high in calories and protein may be beneficial therapeutic options. A randomized, double-blind placebo-controlled pilot trial explored the value of a high-calorie, high-protein supplement in 29 subjects with HFrEF who had experienced an edema-free weight loss of >7.5% over at least 6 months [84]. Subjects were then randomized 3:1 to receive the supplement, which supplied 600 calories and 20 g of protein, or placebo supplying 12 calories with a similar taste and texture for a 6-week period [84]. There was no ET included as part of the intervention [84]. All subjects were instructed to continue their normal diet. At the 6-week end of study visit, Patients in the supplementation group did gain a significant amount of weight, mostly FM on DEXA scan, which was maintained at the 18-week follow-up visit [84]. On DEXA, LM was increased in the supplemented patients at 6 weeks, but this change was not maintained at 18 weeks [84]. While 6MWT distance increased in supplemented patients, relative peak VO_2_ did not increase upon CPET, and LVEF was not increased upon echocardiogram analysis [84]. TNFα was significantly reduced in supplemented patients [84]. Although relative peak VO_2_ did not increase in supplemented subjects versus placebo, suggesting no changes in CRF, the increases in FM and 6MWT distance combined with the decrease in TNFα suggest potential beneficial effects on other important health status indicators in HF, which requires further investigation [108].

### 3.5. Summary of Nutrition Interventions in HFrEF

The supplementation of essential AA, *N*-3 PUFA, and L-carnosine may increase CRF in individuals with HFrEF [77,78,79,81,83] (Table 1). Future work should aim to study the effect of these supplements on CRF over the long term with varied dosing to elucidate the dosing needed to increase CRF. Additionally, biomarkers of intake should be utilized in all future studies to objectively measure intake of the supplements.

### 3.6. Hypocaloric Diets in HFpEF

HFpEF is a heterogenous syndrome characterized by high filling pressures and diastolic abnormalities in the setting of an LVEF ≥50%, which currently lacks evidence-based therapies to reduce morbidity and mortality [109,110,111]. Higher BMI and insulin resistance increase risk for HFpEF more than HFrEF, and over 80% of patients with HFpEF are also overweight and obese [26,112]. This has resulted in the suggestion of a HFpEF-obesity phenotype, although this is controversial, with the opposing argument being that HFpEF and obesity are comorbid conditions with separate CRF abnormalities [103,104,113,114,115]. Lower CRF is linked to worsened prognosis in HFpEF, and ET has proven effective in increasing CRF [116,117]. A trial of 100 patients with obesity and HFpEF randomized subjects into a 20-week 2 × 2 factorial trial consisting of ET, hypocaloric diet (HCD), ET and HCD combined, and attention control groups [87]. ET was individualized and mostly centered on a walking program performed for 1 h, 3 times a week [87]. The HCD was provided by the center’s metabolic kitchen and individualized to the subject’s energy needs, with a 350 calorie deficit for individuals randomized to exercise and caloric restriction and a 400 calorie deficit for those assigned to caloric restriction alone [87]. The attention control received a phone call every 2 weeks from investigators [87]. While both exercise and HCD groups individually demonstrated an increase in relative peak VO_2_ at 20 weeks, the effect of the combined group was additive and resulted in a greater increase in relative peak VO_2_ [87]. Absolute peak VO_2_, not adjusted for body weight, was increased with exercise and unchanged by HCD [87]. Exercise and HCD together also created an additive beneficial effect on peak workload and exercise time, as well as weight loss, which was 10% for HCD and exercise combined, 7% for HCD alone, 3% for exercise alone, and 1% for the control group [87]. On DEXA scan, HCD resulted in loss of LM, FM, and FM percent of body weight, but exercise resulted in loss of FM alone, therefore preserving LM [87]. High sensitivity CRP was reduced by HCD but not exercise, and reduction was correlated with degree of weight lost [87]. Subjects were highly adherent to both exercise (84% sessions attended) and diet interventions (99% from recorded food logs) [87]. Because only small improvements on cardiac mass were found, the authors attributed the increases in exercise capacity to noncardiac peripheral adaptations [87]. This study demonstrated that, particularly in individuals with obesity and HFpEF, HCD and exercise result in larger improvements in CRF than either intervention alone. Furthermore, the increase in relative peak VO_2_ and lack of decrease in absolute peak VO_2_ seen in this trial demonstrate that despite the obesity paradox in HF, which holds that obese patients may have improved survival over their normal and underweight peers [118], the combination of HCD and exercise in individuals with obesity and HFpEF may, in fact, improve CRF. Whether such changes result in improve clinical outcomes requires further study.

### 3.7. Unsaturated Fatty Acid Supplementation in HFpEF

**Figure 2:**
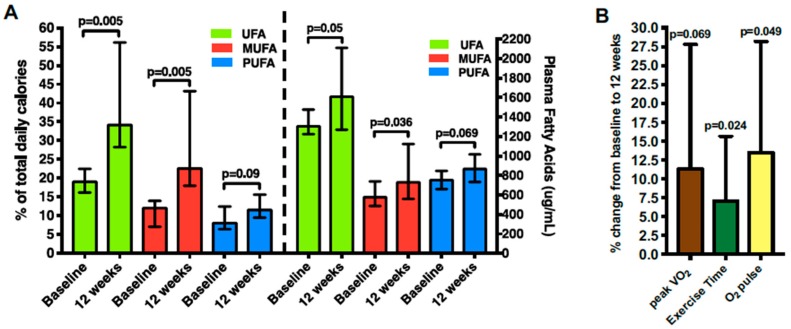
(**A**) Demonstrates increases from baseline to 12-week follow-up in UFA, MUFA, and PUFA expressed in percent total daily calories, as well as plasma fatty acids (μg/mL) in nine HFpEF subjects who supplemented dietary UFA daily for 12 weeks as part of the UFA-Preserved pilot trial. (**B**) Demonstrates trend towards increase in Peak VO_2_ (mL/kg/min), exercise time, and O_2_ pulse (mL/beat) from baseline to 12-week follow-up in the UFA-Preserved pilot trial. Used with permission by Carbone et al. [89]. UFA, unsaturated fatty acids; MUFA, monounsaturated fatty acids; PUFA, polyunsaturated fatty acids; Peak VO_2_, peak oxygen consumption; O_2_, oxygen.

Although a low-fat diet has been long favored in the prevention and treatment of CVD, a diet high in unsaturated fatty acids (UFA) may hold far more cardioprotective benefit [119]. A cross-sectional analysis of 23 subjects with HFpEF and obesity demonstrated that consumption of mono- and polyunsaturated fatty acids (MUFA and PUFA, respectively) was positively correlated with relative peak VO_2_ [88]. This relationship was strengthened when these fatty acids were combined and expressed as total consumption of UFA in grams, as well as in percentage of total calories [88]. Saturated fatty acids were initially also associated with peak VO_2_, but the relationship disappeared upon multivariate analysis when both UFA and saturated fatty acids were included [88]. Dietary sugars were also analyzed and found to be inversely correlated with peak VO_2_ [88]. Additionally, UFA consumption was associated with better diastolic function on echocardiogram, as well as greater FFM percentage of body weight and a better FFM to FM ratio on bioelectrical impedance analysis [88]. In the UFA-Preserved pilot trial, nine subjects with HFpEF and obesity supplemented their diet with UFA-rich foods (including extra-virgin olive oil, canola oil, unsalted or lightly salted mixed nuts, seeds, avocado, and fatty fish) for 12 weeks with baseline dietary counseling and weekly phone call reinforcement by a dietitian [89]. There was no ET included as part of the intervention [89]. After 12 weeks, subjects had a significant increase in UFA plasma biomarkers and reported increased intake of UFA on 24-h dietary recall (Figure 2A) [89]. Subjects demonstrated a trend towards improving their peak VO_2_ on treadmill CPET, which was associated, though non-significantly, with changes in plasma UFA, as well as MUFA and PUFA individually (Figure 2B) [89]. Whether these beneficial effects translate into improvements in clinical outcomes, as well as CRF in a larger sample size, is unknown and requires further work. Currently, the follow-up randomized controlled trial (UFA-Preserved2) is ongoing (NCT03966755) and examining the effect of daily UFA supplementation versus standard of care dietary recommendations (controlling sodium and saturated fatty acids intake) in a randomized, crossover format in 30 subjects with HFpEF and concomitant obesity.

### 3.8. Dietary Approaches to Stop Hypertension in HFpEF

Hypertension is a frequent comorbidity in HFpEF, and a significant portion of individuals with HFpEF may be sensitive to dietary sodium. In a pilot trial of 13 subjects with HFpEF, 21 days of the Dietary Approaches to Stop Hypertension (DASH) diet combined with sodium restriction resulted in an improved distance on the 6MWT, as well as reduced clinic and 24-h (BP) measurements [90]. No ET was included in this intervention [90]. Carotid-femoral pulse wave velocity also significantly improved, and the authors attributed the improvement in 6MWT distance to this reduction in arterial stiffness [90]. Notably, all food was provided for patients during this trial, which likely aided greatly in compliance to the intervention [90]. In 48 patients with HFpEF or HFrEF, 3 months of DASH diet also resulted in improved 6MWT distance versus usual care, although the intervention group failed to reduced sodium consumption [91]. No ET was included as part of the intervention [91]. Endothelial function measured by large and small artery elasticity was not improved in the intervention group versus the control [91]. In this study, the authors argued that the improvement in 6MWT distance may be due more to cardiac and autonomic factors, rather than improvements in endothelial function [91].

### 3.9. Dietary Nitrate Supplementation in HFpEF

Dietary nitrates, which are reduced to NO in the plasma after gut absorption, have been proposed as a mechanism to increase CRF in individuals with HFpEF by decreasing BP and improving SM perfusion. A randomized, placebo-controlled pilot trial examining the effect of dietary nitrates, in addition to aerobic ET, in subjects with HFpEF showed no benefit when compared to placebo on peak VO_2_ [92]. The authors suggested that this may have been related to inadequate dosing; however, a recent trial of inhaled nitrates without ET also failed to increase peak VO_2_ in subjects with HFpEF [93]. Dietary nitrate supplementation has also resulted in mixed effects on CRF in small pilot trials of subjects with HFrEF without ET [85,86]. Currently, the INABLE-Training trial (NCT02713126) is combining higher dosing of oral nitrates with a cardiac rehabilitation (CR) program for 12 weeks in a double-blind, randomized placebo-controlled trial of patients with HFpEF on a primary outcome of peak VO_2_. The study is testing the hypothesis that oral sodium nitrate combined with CR results in greater improvements in peak VO_2_ than CR alone.

### 3.10. Summary of Nutrition Interventions in HFpEF

Evidence-based therapies are urgently needed for HFpEF. With the prevalence of obesity in this population, the combination of ET and HCD is very promising, but would need to be implemented in larger scale, multicenter trials [87] (Table 1). The sodium-restricted DASH diet also offers an appealing treatment for the common comorbidity of hypertension among HFpEF patients and larger, multicenter trials should be implemented [90,91] (Table 1). A home delivery program of the sodium-restricted DASH diet was shown recently to trend towards a reduction in HF 30-day readmissions, demonstrating the possibility that improvements seen in the 6MWT may translate to improved outcomes [120].

## 4. Obesity

Individuals who are less physically active are more likely to become obese, and those who are obese are more likely to avoid PA [121,122,123,124]. Obesity negatively impacts CRF through central and peripheral changes in structure and function, such as remodeling of the heart, restrictive action of excess mass on ventilation, endothelial and mitochondrial dysfunction in the SM, and challenge to the biomechanics of PA [125]. Individuals with obesity, however, do not necessarily have reduced CRF, and “fat but fit” individuals with higher CRF may have attenuated risk for all-cause and CVD mortality, a contradiction known as the obesity paradox [125,126]. Peak VO_2_ is often reported corrected for body mass (relative peak VO_2_), which results in an automatically lowered appearance for individuals with obesity versus their normal weight peers, but CRF may, in fact, be normal or mildly reduced when reported in absolute terms [125]. Nevertheless, obesity remains a major risk factor for numerous noncommunicable disease states, and weight loss is recommended to lower this risk [127]. Weight loss interventions focused solely on diet, however, often result in loss of LM, as well as FM, and it is important to examine the effects this may have on CRF [96,97,98]. This is especially important as weight regain often occurs and the individual may regain to their former weight with more FM and less LM, which is perhaps even more detrimental to their CRF.

### 4.1. Hypocaloric Diets in Obesity

Multiple studies have examined the effects of HCD alone versus HCD combined with ET in individuals with obesity on cardiometabolic risk factors, including CRF [96,97,98,99,128]. Though the studies differed in design, studies that compared HCD alone versus HCD combined with ET found that relative [96,97], relative to LM [128], and sometimes absolute [99] peak VO_2_ increased more in the HCD and ET combination groups versus the HCD alone. One study was unclear on whether an absolute or relative peak VO_2_ was being reported, but peak VO_2_ was still reported as increased more in the HCD and ET group versus HCD alone [98]. Body weight and FM loss was usually similar in the HCD and ET groups versus HCD alone [96,97,98,128], but this was not always the case [99]. HCD only groups often experienced a greater loss of LM or LM as a percent of body weight [96,97,98,99], but not always [97]. Loss of waist circumference, reflecting visceral adiposity, was sometimes greater in the ET and HCD groups versus HCD alone [99,128], but not always [97].

### 4.2. Macronutrient Composition of Hypocaloric Diets in Obesity

Although recent studies have suggested that macronutrient composition of HCD does not affect the degree of weight lost [18], the question remains of whether it affects CRF. One randomized study examined the effects of an extremely low carbohydrate versus a moderately low-fat diet in 60 men and women who were overweight or obese after 8 weeks of a 52-week intervention [129]. Subjects also had abdominal obesity and at least one metabolic syndrome risk factor, as defined by the AHA [129]. The diets were equally hypocaloric and the low carbohydrate HCD consisted of 35% energy from protein, 61% from fat, and 4% from carbohydrate, while the low-fat HCD had 24% energy from protein, 30% from fat, and 46% from carbohydrate [129]. There was no ET component to the intervention [129]. Participates met with a dietitian and turned in semi-quantitative food records every 2 weeks, and compliance to the low carbohydrate diet was measured with levels of plasma ketones [129]. At the end of the 8 weeks, men lost more weight, as well as FM, measured by DEXA on the low carbohydrate HCD, while a difference in weight and FM loss between the two HCD was not observed in women [129]. On treadmill CPET, absolute peak VO_2_ was reduced and relative peak VO_2_ remained the same in both groups, with no effect of HCD [129]. For the 43 participants who completed the entire 52-week period of the study, both groups had similar reductions in weight and FM loss [130]. Absolute peak VO_2_ remained decreased from baseline, but relative peak VO_2_ increased in both groups, without differences between groups [130]. Another randomized study examined altering the protein composition of a low-fat HCD in 56 overweight or obese men for 12 weeks [131]. There was no ET included as part of the intervention [131]. The diets were moderately hypocaloric and the high protein HCD consisted of 35% energy from protein, 40% from carbohydrate, and 25% energy from fat, while the low protein HCD provided 17% energy from protein, 58% energy from carbohydrate, and 25% energy from fat [131]. Participants met with a dietitian and turned in semi-quantitative food records every 2 weeks during the intervention [131]. Weight and FFM loss were not different between the two groups, but there was a non-significant trend towards greater FM loss in the high protein group [131]. On treadmill CPET, absolute peak VO_2_ did not change, but relative peak VO_2_ increased, with no between HCD group differences [131].

### 4.3. Summary of Nutrition Interventions in Obesity

In determining optimum plan for weight loss in obesity, it appears that it may be slightly more beneficial to add exercise to a HCD in terms of reduction in visceral adiposity [99,128] and preventing a loss of LM [96,97,98,99]. Total weight and FM loss do not seem to differ, regardless of whether or not a exercise component is added to a HCD [96,97,98,128]. Effects on absolute peak VO_2_ are mixed [99,129,130], but relative peak VO_2_ is likely to stay the same or increase [96,97,128,130,131]. Macronutrient composition of the HCD does not seem to matter on weight, LM, or FM loss, or peak/absolute VO_2_ change, but more research is clearly needed to definitively answer this important question [129,130,131]. Overall, short-term weight loss is likely not harmful and may be beneficial to CRF; however, longer term follow-up is needed to determine the potential difference in outcomes in those who regain compared to those who do not regain weight post-intervention.

## 5. Sarcopenia and Frailty

Sarcopenia is a reduction in SM strength accompanied by either a loss of mass or quality while FM is preserved [132]. When low physical performance accompanies these characteristics, sarcopenia is considered severe [132]. Sarcopenia affects 5–20% of the population above 60 and up to 50% of individuals over 80 years of age [27]. Individuals with sarcopenia have a reduced CRF compared to their non-sarcopenic peers, especially in the presence of high FM, a condition known as sarcopenic obesity [133,134,135,136]. Furthermore, in individuals with HF, sarcopenia may be even more detrimental to CRF and quality of life than cachexia [135]. Frailty overlaps heavily with the physical manifestations of sarcopenia, but encompasses a broader range of geriatric decline that also includes cognitive and social deterioration associated with adverse outcomes [132].

### 5.1. Nutrition Interventions in Sarcopenia and Frailty

Most nutrition interventions in individuals with sarcopenia and frailty have included physical performance measures which attempt to gauge the effect of both SM and nervous system function on locomotion rather than CRF. These measures include the short physical performance battery (SPPB), timed-up and go test, gait speed, and occasionally the 400 m walk. The 400 m walk, like the 6MWT or ISWT, attempts to estimate CRF without the expense, trained personnel, and time required for CPET [137]. Many nutrients have been proposed to address the key physical components (lack of muscle strength, mass, quality, low physical performance) present in sarcopenia, including increased protein intake, creatine, Leucine, β-hydroxy- β- methylbutyrate (HMB), vitamin D, and *N*-3 PUFA [138]. As studies remain heterogenous and large clinical trials are still lacking, no firm recommendations regarding nutrition interventions for sarcopenia and frailty currently exist, though essential AA and HMB have shown an effect on SMM and function [138,139].

### 5.2. Dietary Patterns in Sarcopenia and Frailty

Outside of individual nutrients, healthy dietary patterns may help to lessen the severity or delay onset of physical performance and CRF decline in older adults. In a cross-sectional analysis of 380 Spanish adults aged 55–80, higher adherence to a traditional Mediterranean dietary pattern was associated with greater walking distance on the 6MWT, while higher adherence to a Western dietary pattern was associated with a lower 6MWT distance [140]. In 117 individuals with T2DM, those over 75 years of age and in the highest tertile of adherence to the Mediterranean Diet demonstrated greater 6MWT and 10-min walk test distances than those less adherent [141].

### 5.3. Protein Supplementation in Sarcopenia and Frailty

Currently, there are few nutrition interventions in older adults with physical characteristics of sarcopenia or frailty that include CRF measurements. In 80 older adults aged 70–85 with a low SPPB score (<10), a 6-month randomized, double-blind placebo-controlled trial of whey protein was conducted in combination with regular resistance ET [94]. Participants received 40 g of whey protein split into two doses per day or an isocaloric amount of maltodextrin [94]. Adherence to the nutrition intervention was measured by an objective biomarker, para-aminobenzoic acid, which was added to the supplements [94]. The supervised resistance ET program took place 3 times a week [94]. Whey protein has been theorized to be an ideal supplement to promote SM anabolism as it has a high proportion of essential AA [94]. Participants improved in strength in all muscle groups measured and gained some LM; however, there were no differences between groups and there was no significant change in 400 m walk distance from baseline [94]. Another recent trial randomized 149 participants over 70 years of age with low vitamin D status and SPPB score (≤9) to a ready-to-drink supplement containing whey protein and vitamin D versus a low calorie, non-nutritive beverage for 6 months [95]. Participants also underwent a PA program 3 days a week for the duration of the trial [95]. The supplement contained 150 calories, 20 g of whey protein, and 800 international units of vitamin D, while the identical placebo contained 30 calories [95]. While the supplement group experienced greater declines in intramuscular fat measured by computed tomography than the placebo group, both groups had similar decreases in total body FM on DEXA scan and gains in muscular strength [95]. There were also improvements in both groups in the 400 m walk test, but no between-group differences were observed [142].

### 5.4. Summary of Nutrition Interventions in Sarcopenia and Frailty

Although individuals with sarcopenia have a low CRF compared to their nonsarcopenic peers [133,134,135,136], there are a lack of trials currently that demonstrate efficacy of nutrition interventions in altering CRF in this population, despite increases in CRF at the lower end of the spectrum having the greatest effect on health outcomes [1] (Table 1). Future trials should also focus on recruiting subjects with sarcopenia defined by population-appropriate guidelines [132,143].

## 6. Future Directions

As recognition of low CRF as a risk factor for morbidity and mortality grows, more knowledge is accrued about the pathogenesis of exercise intolerance in different disease states and CRF is more widely used as an outcome measure. A wide variety of disease states such as chronic kidney disease (CKD), cancer, non-alcoholic steatohepatitis, atrial fibrillation, and pulmonary hypertension are now recognized to be associated with reduced CRF, and all have a role for nutrition therapy that may assist in increasing CRF and improving outcomes [144,145,146,147,148,149].

Exercise testing in subjects with stage 3–4 CKD has revealed a reduced CRF with altered central and peripheral responses to exercise compared to healthy age matched controls [144]. Furthermore, in subjects with stage 2–4 CKD who performed the 6MWT, those who walked a distance below 350 m versus those above that threshold had a significantly decreased risk of survival [145]. In terms of dietary changes and their effects on CRF in individuals with CKD, little is known. A recent trial randomized 111 subjects with CKD with overweight/obesity in a 2 × 2 factorial trial to caloric restriction, ET, both caloric restriction and ET, or usual care [150]. Mean glomerular filtration rate was 41 ± 18.6 mg/mL per 1.73 m^2^ [150]. After 4 months, there were no evident effects of the interventions on peak VO_2_ [150]. Further research is greatly needed regarding the effects of nutrition on CRF in CKD and a wealth of dietary targets exist [151].

Individuals with cancer and cancer survivors are another group more recently recognized to have a reduced CRF. Aging-related declines in CRF, coupled with the effects of cancer treatments (radiation, chemotherapy, hormone therapy etc.), can lead to significantly reduced exercise tolerance [146]. Several recent trials in cancer survivors have found that lifestyle interventions combining dietary counseling and PA/ET have been able to improve relative peak VO_2_ compared to usual care [152,153] and in comparison to subjects’ baseline [154]. It is difficult to distinguish the effects of ET from nutrition-related improvements, as trials thus far have not included separate ET and diet intervention groups. This is particularly relevant, as these trials reported CRF as relative peak VO_2_ and also reported decreases in weight, in which dietary intervention may play a larger role [152,153,154].

## 7. Limitations

Limitations of trials aimed at examining the effects of nutrition on CRF are notable and widespread in many nutrition research studies. Dosing of nutrition supplements, diet design, and approaches to nutrition counseling differ widely between studies [155]. Trials are often small and obviously difficulties in blinding exist, especially in regard to dietary patterns [155]. Measuring adherence to the dietary intervention is often much more challenging than measuring pharmaceutical adherence. Developing standards of practice and design in nutrition research remains an ongoing issue. However, increasing recognition and calls to action to increase the rigor of nutrition research will hopefully result in trials that more definitively provide guidelines for evidence-based practice, including in the use of nutrition therapy to increase CRF [155].

## 8. Conclusions

Nutrition therapy offers a promising role in increasing CRF in multiple populations with exercise limitations, including COPD, HF, obesity, sarcopenia, and frailty. As disease states with reduced CRF continue to be defined, nutrition intervention trials in these populations should include measurements of CRF. Additionally, the potential effect of dietary patterns such as DASH and Mediterranean diets on CRF should be studied in a randomized format in noncommunicable disease states where they have shown improvements in other surrogate measures of health. Standardized approaches and larger trials are clearly needed to determine which therapies may be the most efficacious and result in improvements in clinical outcomes.

## Figures and Tables

**Table 1:** A summary of the lifestyle interventions with a nutrition component described in this paper in chronic obstructive pulmonary disease (COPD), heart failure (HF), sarcopenia, and frailty. As obesity focuses primarily on weight loss interventions, interventions have been summarized separately in Table 2.

	Peak VO_2_	6 MWT	ISWT	400 m Walk
**COPD**				
Creatine [35,36,37]	↔	??	↔	
*N*-3 PUFA [38]	↔	??	??	
Vitamin D [43]	??	??	??	
Vitamin B12 [44]	??	??	??	
Dietary Nitrates [39,40,41,63,64]	↔	↔	↑	
Nutrition Support Supplements [45,46,47,49,50]	??	↔	↔	
**HFrEF**				
Multivitamin [76]	??	↔		
Essential AA [77,78,79]	↑	↑		
BCAA [80]	↔	??		
L-Carnosine [81]	↑	↑		
Glutamine + *N*-3 PUFA [82]	↔	↔		
*N*-3 PUFA [83]	↑	↔		
Nutrition Support Supplements [84]	↔	↑		
Dietary Nitrates [85,86]	↔	??		
**HFpEF**				
HCD [87]	↑	??		
UFA Supplementation [88,89]	??	??		
DASH Diet [90,91]	??	↑		
Dietary Nitrates [92,93]	↔	??		
**Sarcopenia and Frailty**				
Whey Protein [94]	??	??		↔
Whey Protein + Vitamin D [95]	??	??		↔

HCD, Hypocaloric diet; peak VO2, peak oxygen consumption; 6MWT, six minute walk test; ISWT, incremental shuttle walking test; COPD, chronic obstructive pulmonary disease; HFrEF, heart failure with reduced ejection fraction; HFpEF, heart failure with preserved ejection fraction; *N*-3 PUFA, omega 3 polyunsaturated fatty acids; AA, amino acids; BCAA, branch chain amino acids; UFA, unsaturated fatty acids; DASH, Dietary Approach to Stop Hypertension; ↑ Increase, ↔ No Effect, ?? Lack of Data at this time.

**Table 2:** Comparison between diet, exercise, and diet and exercise combined in published randomized controlled trials. The combination of hypocaloric diet (HCD) and exercise training (ET) has demonstrated more consistent improvements in peak oxygen consumption (VO_2_) than ET or diet alone [96,97,98,99].

	Peak VO_2_	Units
**Diet**		
Villareal et al. (2011)	↑	ml/kg/min
Nicklas et al (2009)	↑	ml/kg/min
Straznicky et al (2010)	↔	ml/kg_FFM_/min
Foster-Schubert et al (2012)	↔	L/min
**Exercise**		
Villareal et al. (2011)	↑	ml/kg/min
Nicklas et al. (2009)	N/A	ml/kg/min
Straznicky et al. (2010)	N/A	ml/kg_FFM_/min
Foster-Schubert et al. (2012)	↑	L/min
**Diet and Exercise**		
Villareal et al. (2011)	↑↑	ml/kg/min
Nicklas et al. (2009)	↑↑	ml/kg/min
Straznicky et al. (2010)	↑↑	ml/kg_FFM_/min
Foster-Schubert et al. (2012)	↑	L/min

HCD, Hypocaloric diet; peak VO2, peak oxygen consumption; ml/kg/min, milliliters of oxygen consumption per minute; ml/kg_FFM_/min, milliliters of oxygen consumption per kilogram fat free mass per minute; L/min, liters of oxygen consumption per minute; ↑ Increase from baseline, ↑↑ Increase versus both exercise and diet intervention, ↔ No effect seen.
